# Demographic expansion of two *Tamarix* species along the Yellow River caused by geological events and climate change in the Pleistocene

**DOI:** 10.1038/s41598-017-19034-x

**Published:** 2018-01-08

**Authors:** Hong-yan Liang, Zhi-pei Feng, Bing Pei, Yong Li, Xi-tian Yang

**Affiliations:** 1 College of Forestry, Henan Agricultural University, Zhengzhou, 450002 China; 2 Sanmenxia Polytechnic, Sanmenxia, 472000 China

## Abstract

The geological events and climatic fluctuations during the Pleistocene played important roles in shaping patterns of species distribution. However, few studies have evaluated the patterns of species distribution that were influenced by the Yellow River. The present work analyzed the demography of two endemic tree species that are widely distributed along the Yellow River, *Tamarix austromongolica* and *Tamarix chinensis*, to understand the role of the Yellow River and Pleistocene climate in shaping their distribution patterns. The most common chlorotype, chlorotype 1, was found in all populations, and its divergence time could be dated back to 0.19 million years ago (Ma). This dating coincides well with the formation of the modern Yellow River and the timing of Marine Isotope Stages 5e-6 (MIS 5e-6). Bayesian reconstructions along with models of paleodistribution revealed that these two species experienced a demographic expansion in population size during the Quaternary period. Approximate Bayesian computation analyses supported a scenario of expansion approximately from the upper to lower reaches of the Yellow River. Our results provide support for the roles of the Yellow River and the Pleistocene climate in driving demographic expansion of the populations of *T. austromongolica* and *T. chinensis*. These findings are useful for understanding the effects of geological events and past climatic fluctuations on species distribution patterns.

## Introduction

The uplift of the Qinghai-Tibet Plateau (QTP) dramatically changed the topography of Asia, and the direction of flow of the Yellow River system from west to east is closely tied to this uplift. The Yellow River appeared about 1.7 Ma during phase C of the Qingzang Movement^1^. 1.2 Ma before present, there was substantial uplift of the Tibetan Plateau^2^, and tectonic movement of the Kunlun-Yellow River caused the Yellow River to cut the Jishi Gorge and flow into the Linxia-Lanzhou basin^3^. At that time, the Yellow River basin was only composed of some disconnected lakes^4^. The uplift of the Tibetan Plateau and river erosion, especially during the Gonghe Movement (0.15 Ma) in the late Pleistocene, greatly promoted the connection of these lakes causing the retrogressive erosion of the Yellow River and downcutting of the river valley in Sanmen Gorge^5^. Consequently, the Yellow River evolved into a long river flowing from west to east^6^. The river might have acted as an abiotic physical barrier to gene flow^7,8^, or it may have geographically facilitated movement and channels of dispersal, which has been observed in *Rosa roxburghii*^9^, *Rhododendron ripense*^10^ and *Terminalia franchetii*^11^, for which frequent gene flow resulted in the genetic homogenization of populations.

The Pleistocene was a time during which dramatic climatic and temperature shifts occurred^12^. This was especially true during the Mid-Pleistocene Transition (MPT), which lasted from approximately 1.25 million to 700 thousand years ago, and was a period during which the total amount of ice in ice sheets increased globally^13,14^. The climatic fluctuation in China during the Pleistocene, although not as severe as that in Europe and North America, still occurred during the alternation between glacial and interglacial periods^15,16^. In China, loess sequences in central China indicated that major shifts occurred toward cooler and drier climates at about 2.4 Ma, 1.2 Ma, and 0.5 Ma, while less pronounced shifts were dated to 1.65 Ma, 0.8 Ma, and 0.2 Ma^17,18^. Furthermore, marine δ^18^O records also showed similar shifts^19^. Three ice core records from the Qinghai-Tibet Plateau revealed that late glacial stage conditions were apparently colder, wetter, and dustier than Holocene conditions^20^. Additionally, lacustrine sporopollen records indicated that wetter conditions progressed from western China to the east during the early Pleistocene^21^. These climatic changes can influence the distribution of species because the distributions of some species migrated during glacial periods, and the species survived in refugia, after which many species then re-colonized some areas during the postglacial period^22–24^; alternatively, other species persisted and adapted to the changed conditions^25,26^, while others became extinct^27^.

*Tamarix austromongolica* and *Tamarix chinensis* are endemic tree species in China^28^; *T. austromongolica* is naturally distributed in the upper reaches of the Yellow River, from Qinghai to the ravine region between Shanxi and Shaanxi provinces; *T. chinensis*, however, mainly occurs in the lower reaches of the Yellow River. The most obvious features of these species include drought, saline, and alkaline tolerance; they are also water tolerant. The small, short-lived seeds in particular require a moist surface soil, short-term precipitation, or may drift with water in the early stage of germination^29^, which is different from other desert shrubs. As two species that are widely distributed along the coast of the entire Yellow River, *T. austromongolica* and *T. chinensis* are very closely related species^30^. Furthermore, *Tamarix* is an ancient genus occurring in ancient times in the Mediterranean region^31^. Therefore, these two species can serve as good candidates for investigating the influence of the Yellow River and paleoclimate on patterns of species distribution.

In this study, we sampled 45 populations along the Yellow River to infer the effects of geological events and climate change during the Pleistocene on two *Tamarix* species. To better understand the distribution of these two species, we evaluated the following: (1) the relationship between the divergence time of chlorotypes and the paleoclimate, formation, and evolution of the Yellow River; (2) the demographic history of two *Tamarix* species using genetic data and species distribution models.

## Results

### Geographic distribution and network structure of haplotypes

**Figure 1:**
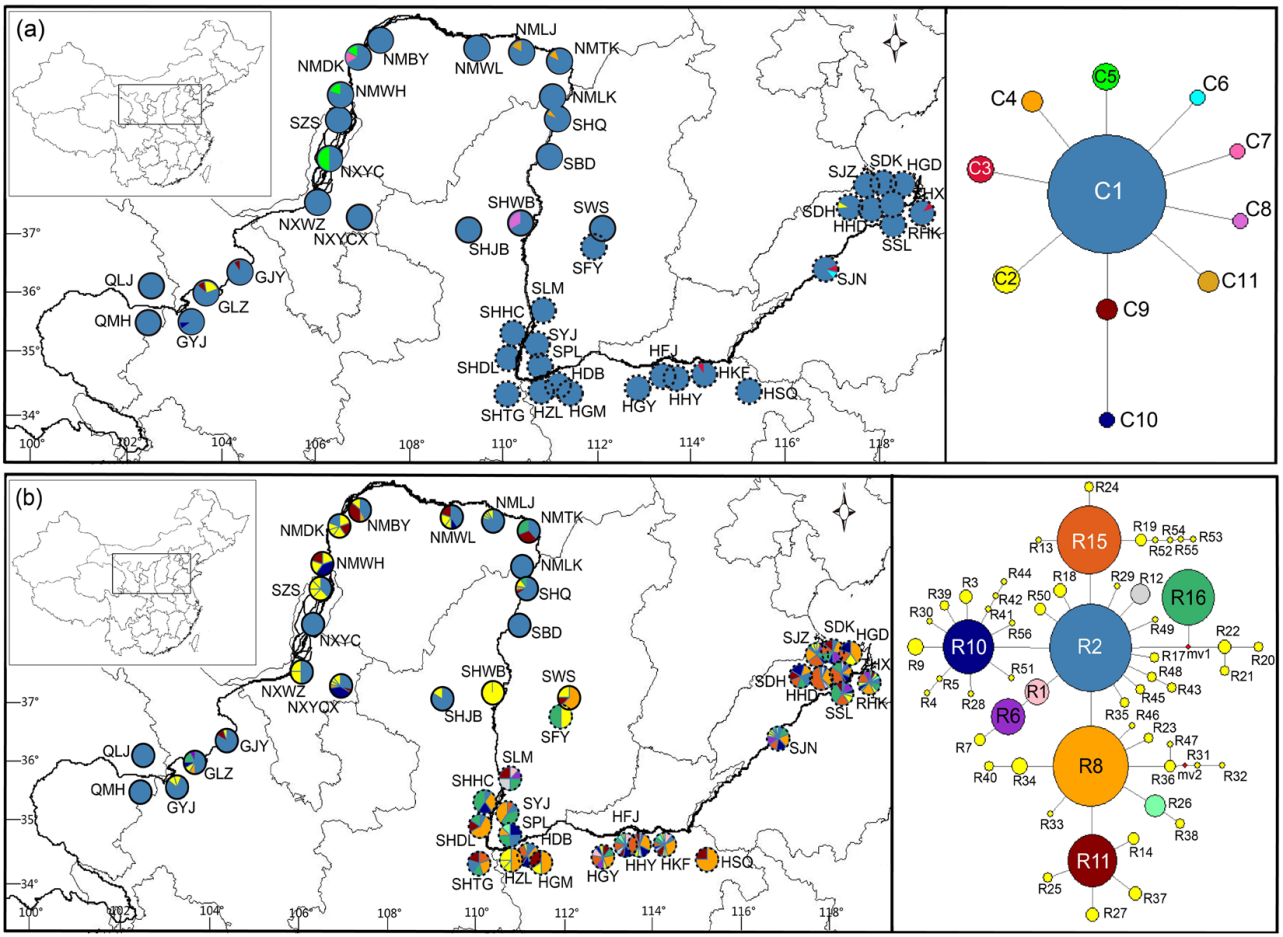
Detailed sampling locality information, chlorotype distribution (**a**), and ribotype distribution (**b**) of *T. austromongolica* (solid circles) and *T. chinensis* (dotted circles). The color of the pie chart corresponds to the haplotype in the median-joining network. The size of the circles corresponds to the frequency of each haplotype. The base map was downloaded from DIVA-GIS (http://www.diva-gis.org/gdata). The figure was drawn using Diva-GIS v7.5.0 (http://www.diva-gis.org/) and Adobe Illustrator CS5 v15.1.0 (Adobe Systems, Inc.).

Two cpDNA fragments from a total of 382 individuals and nDNA ITS fragments of 45 populations and two out-group individuals were sequenced. The total length of cpDNA sequences was 1640 bp, with 11 chlorotypes identified by nine nucleotide substitution sites and two indels. These 11 chlorotype sequences were submitted to the GenBank database under accession numbers KY621817-KY621842. Chlorotype C10 was unique to population GYJ. The Chlorotype C1 was shared by all of populations, which indicates high migration rates among populations. Interestingly, C2 was found in populations GLZ and SDH, in different species and at different geographical distance between the two populations. The details of chlorotype distribution for each locality are shown in Fig. 1a.

Based on chlorotypes, we constructed a Median-joining network. One chlorotype (C1) was found in 363 of 382 individuals from the 45 populations and represented the central node of this network; the networks containing clades with characteristic star-like topologies showed that populations that have experienced population expansion. The aligned sequences of ITS in 45 populations were 686 bp; 56 ribotypes (see Fig. 1b) were found among 363 individuals (a total of 726 alleles). Ribotypes R2, R8, R15, R11, R10, and R16 contained a large number of haplotypes; the sequence polymorphisms detected in cpDNA and ITS regions are shown in Supplementary Tables S1, S2.

### Genetic diversity and genetic structure

The haplotype and nucleotide diversities inferred from cpDNA sequences show that a low level of variation existed among the populations. Haplotype diversity, Hd, ranged from 0 to1; and nucleotide diversity, π, within populations ranged from 0 to 0.61 × 10^−3^ (Table 1).

**Table 1:** Geographical and genetic information inferred from cpDNA sequences for the sampled populations of *Tamarix austromongolica* and *Tamarix chinensis*.

Location	Lat. (N)/Long. (E) (°)	Elev. (m)	Chlorotypes (no. of individuals)	Hd	π × 10^−3^	Location	Lat. (N)/Long. (E) (°)	Elev.(m)	Chlorotypes (no. of individuals)	Hd	π × 10^−3^
*T. austromongolica*											
Minhe, QMH	35.51/102.55	1792	C1(6)	0	0	Bayannaoer, NMBY	40.40/107.22	1039	C1(10)	0	0
Lijia, QLJ	36.12/102.57	1794	C1(5)	0	0	Wulate, NMWL	40.18/109.53	1064	C1(7)	0	0
Yongjing, GYJ	35.57/103.14	1728	C1(9)C10(1)	0.20	0.24	Liangjiagedu, NMLJ	40.12/110.38	1032	C1(10)C11(2)	0.30	0.19
Lanzhou, GLZ	36.03/103.48	1520	C1(6)C2(2)C9(1)	0.56	0	Tuoketuo, NMTK	40.11/111.12	1056	C1(7)C4(1)	0.25	0.15
Jingyuan, GJY	36.35/104.42	1421	C1(10)C9(1)	0.18	0	Longkou, NMLK	39.30/111.14	1324	C1(6)	0	0
Yanchi, NXYCX	37.31/107.04	1527	C1(10)	0	0	Hequ, SHQ	39.22/111.06	860	C1(11) C4(1)	0.17	0.10
Wuzhong, NXWZ	37.59/106.09	1127	C1(7)	0	0	Baode, SBD	39.01/111.03	816	C1(5)	0	0
Yinchuan, NXYC	38.30/106.33	1130	C1(1)C5(1)	1.00	0.61	Wenshui, SWS	37.27/112.14	703	C1(2)	0	0
Shizuishan, SZS	39.13/106.46	1100	C1(6)	0	0	Jingbian, SHJB	37.21/109.10	1522	C1(5)	0	0
Wuhai, NMWH	39.39/106.46	1082	C1(4)C5(1)	0.40	0.24	Wubao, SHWB	37.26/110.41	645	C1(2) C8(1)	0.67	0.41
Dengkou, NMDK	40.18/107.01	1053	C1(4)C5(1)C7(1)	0.60	0.41						
*T. chinensis*											
Fengyang, SFY	37.06/112.11	884	C1(5)	0	0	Huayuankou, HHY	34.54/113.39	83	C1(15)	0	0
Longmen, SLM	35.39/110.36	393	C1(4)	0	0	Kaifeng, HKF	34.54/114.20	84	C1(15) C3(1)	0.13	0.08
Hechuan, SHHC	35.09/110.20	352	C1(4)	0	0	Shangqiu, HSQ	34.34/115.41	60	C1(4)	0	0
Yongji, SYJ	34.50/110.15	334	C1(5)	0	0	Jinan, SJN	36.43/117.00	40	C1(28)C3(1)C6(1)	0.13	0.08
Dali, SHDL	34.45/110.13	340	C1(6)	0	0	Donghuang, SDH	37.51/117.54	3	C1(9) C2(1)	0.20	0
Tongguan, SHTG	34.36/110.17	325	C1(7)	0	0	Huanghedao, HHD	37.55/118.01	3	C1(9)	0	0
Pinglu, SPL	34.50/111.20	318	C1(7)	0	0	Zhanhua, ZHX	37.44/118.04	3	C1(10)	0	0
Zhongliu, HZL	34.49/111.20	288	C1(4)	0	0	Diaokou, SDK	38.01/118.43	1	C1(14)	0	0
Sanmenxia, HDB	34.49/111.21	289	C1(21)	0	0	Sanjiaozhou, SJZ	38.03/118.40	1	C1(10)	0	0
Gaomiao, HGM	34.47/111.16	296	C1(3)	0	0	Dongying, HGD	37.53/118.43	5	C1(5)	0	0
Gongyi, HGY	34.48/113.05	185	C1(8)	0	0	Shengliqiao, SSL	37.36/118.32	6	C1(7)	0	0
Zhengzhou, HFJ	34.57/113.30	96	C1(13)	0	0	Ruhaikou, RHK	37.45/119.09	1	C1(17) C3(1)	0.11	0.07

Note: Hd, haplotype diversity; π, nucleotide diversity within population; Elev., elevation; Lat., latitude; Long., longitude.

Both chlorotypes and nuclear ribotypes failed to reflect obvious phylogeographic structure using the STRUCTURE program. CpDNA analyses of molecular variance (AMOVA) indicated that only 0.80% of this variation was observed among *T. austromongolica* and *T. chinensis* (Table 2), whereas 96.26% of the variance was intra-populational. For ITS dataset, the AMOVA showed that 75.46% of the variance was within populations.

**Table 2:** Hierarchical analysis of molecular variance on *Tamarix austromongolica* and *Tamarix chinensis*. Note: d.f., degrees of freedom.

Variance component	d.f.	Percentage of variation (%)	Φ-statistics	*P* Value
cpDNA				
Among two groups	1	0.80	ΦCT = 0.008	<0.01
Among populations	43	2.94	ΦSC = 0.029	>0.05
Within populations	337	96.26	ΦST = 0.037	=0.05
nDNA				
Among two groups	1	17.24	ΦCT = 0.172	<0.01
Among populations	43	7.31	ΦSC = 0.088	<0.01
Within populations	681	75.46	ΦST = 0.245	<0.01

### Time of divergence for chlorotypes

**Figure 2:**
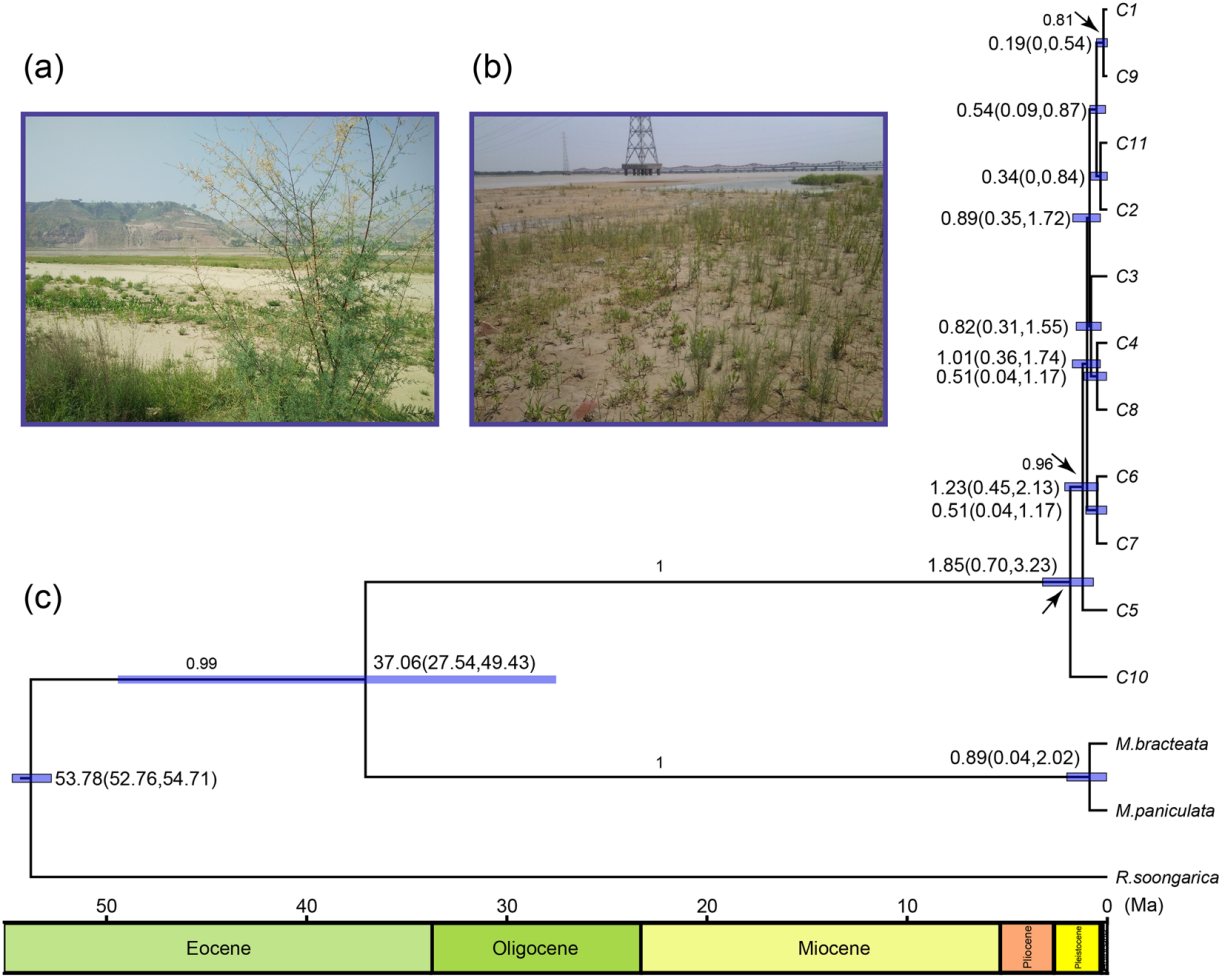
(**a**) *Tamarix austromongolica* at site SHQ; (**b**) *Tamarix chinensis* at site HFJ; (**c**) BEAST-generated maximum clade credibility tree of 11 chlorotypes. The length of the blue bars represents 95% highest posterior density, and posterior probabilities are given above the main branches.

In the time-calibrated chlorotype tree (Fig. 2), all chlorotypes diverged during the Pleistocene. The BEAST analyses placed the origin of *T. austromongolica* and *T. chinensis* at 1.85 Ma (95% HPD = 0.70–3.23), which was observed in the GYJ population in this study, suggesting that plants of the genus *Tamarix* first reached Gansu when migrating from a secondary differentiation center of Xinjiang. The divergence time of C5 (found in NMDK, NMWH, and NXYC) was 1.23 Ma (95% HPD = 0.45–2.13). Chlorotype C1 is the most widely distributed in the Yellow River basin, and its divergence was 0.19 Ma (95% HPD = 0–0.54), which is approximately consistent with the time of MIS 5e-6 and formation of the modern Yellow River, suggesting that climate fluctuations promoted the divergence of chlorotypes of *Tamarix*.

### Demographic analyses

Neutrality tests detected that populations of *T. austromongolica* have experienced recent demographic expansion based on cpDNA sequences (Table 3). In general, the significantly negative Tajima’s D and Fu’s F values were interpreted as a signal of purifying selection or alternately as demographic expansion. The values of Tajima’s D and Fu’s F inferred from nDNA were negative but statistically insignificant.

**Table 3:** Demographic estimates for all samples of *Tamarix austromongolica* and *Tamarix chinensis. ns*: not significant, *P < 0.05. Note: SSD, sum of squares deviations.

Species	cpDNA				nDNA			
	SSD (p)	Raggedness index (p)	Tajima’s D	Fu’s Fs	SSD (p)	Raggedness index (p)	Tajima’s D	Fu’s Fs
*T. austromongolica*	0.009(0.043)	0.161(0.343)	−0.273*	−0.03*	0.026(0.279)	0.16(0.456)	−0.306^*ns*^	0.135^*ns*^
*T. chinensis*	0(0.023)	0.089(0.159)	−1.165^*ns*^	−0.794^*ns*^	0.065(0.185)	0.244(0.336)	−0.039^*ns*^	−0.988^*ns*^
Total	0.005(0.037)	0.123(0.246)	−0.213*	−0.107*	0.047(0.228)	0.205(0.389)	−0.164^*ns*^	−0.464^*ns*^

The mismatch distributions of both cpDNA and nDNA revealed the occurrence of an expansion of the historical population (Table 3). Sum of squares deviations (SSD) tested the validity of a sudden expansion model based on the SSDs between the observed and expected mismatch. Non-significant values for SSDs indicated that the data did not deviate from that expected under the model of expansion. The raggedness index was calculated similarly, and insignificant raggedness values also indicated populations of both species experienced expansion.

**Figure 3:**
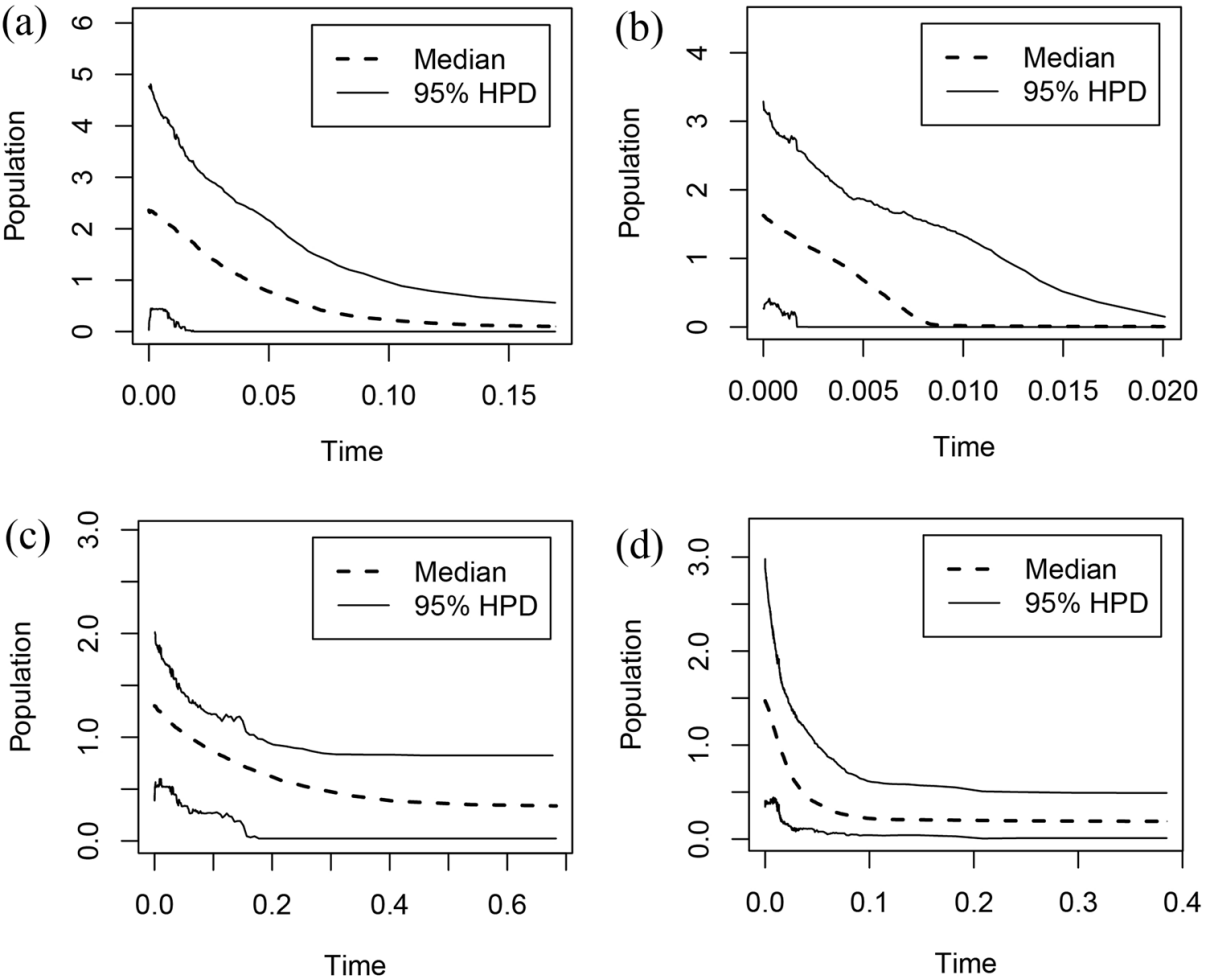
Past demographic history based on (**a**) samples of cpDNA from *Tamarix austromongolica*; (**b**) samples of cpDNA from *Tamarix chinensis*; (**c**) ITS samples from *T. austromongolica*; and (**d**) ITS samples from *T. chinensis* estimated using extended Bayesian skyline plots. In each plot, the x-axis represents the time before present (Ma) and the y-axis represents the effective population size. The dashed and solid lines indicate the median and 95% highest posterior density intervals, respectively.

The Extended Bayesian Skyline Plot (EBSP) of *T. austromongolica* cpDNA indicated a continuous demographic expansion that began around 0.10 Ma (Fig. 3a), and the historical population trend inferred from ITS also showed that *T. austromongolica* experienced a long period of steady and sustainable growth in its populations (Fig. 3c). The EBSP of *T. chinensis* cpDNA showed an initial period of stable size followed by an abrupt period of growth in effective population size (Fig. 3b); this change of the effective population size of *T. chinensis* was also observed in the ITS data sets (Fig. 3d).

### Evolutionary path of *T. austromongolica* and *T. chinensis* along the Yellow River

**Figure 4:**
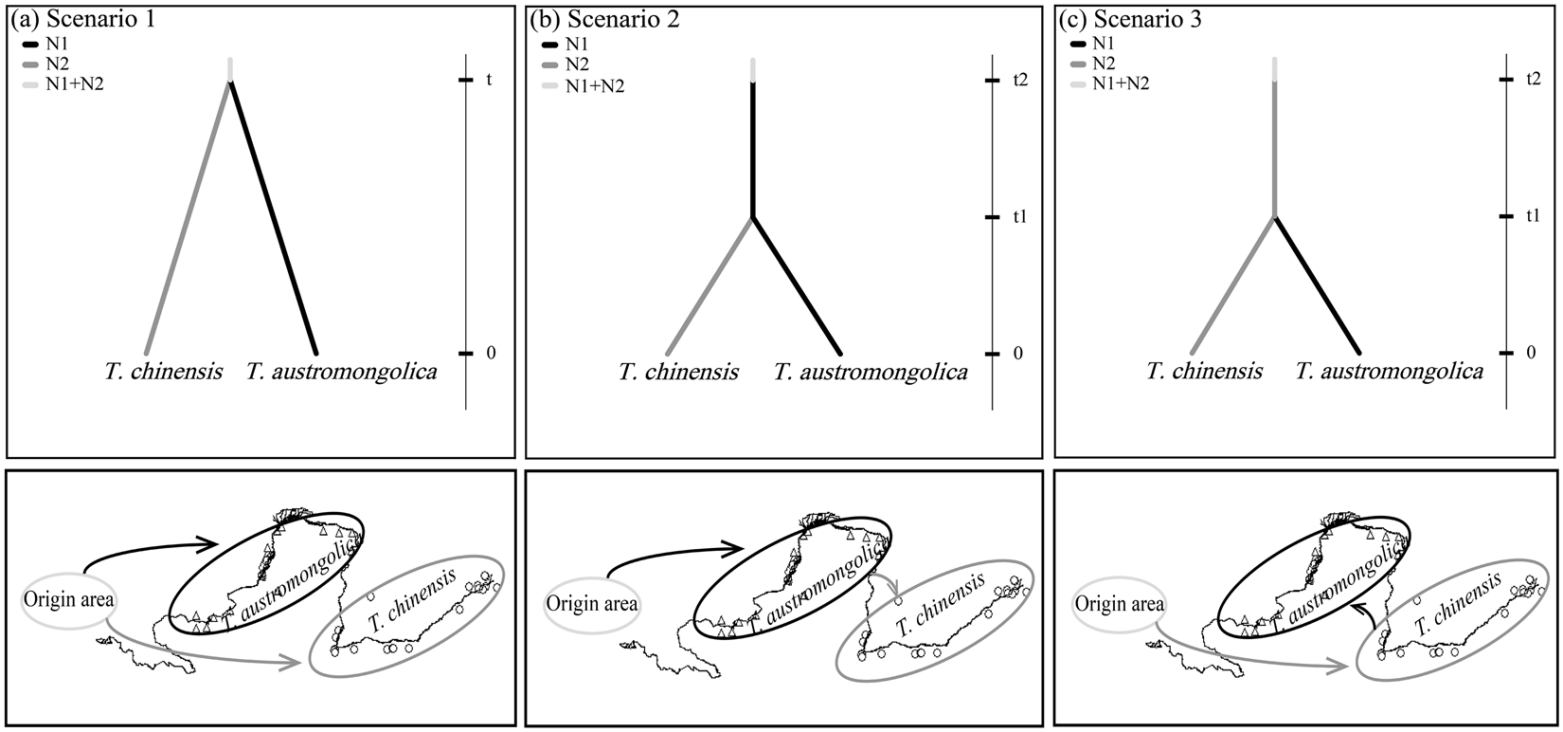
Scenarios of *Tamarix austromongolica* and *Tamarix chinensis* divergence. (**a**) scenario 1; (**b**) scenario 2; (**c**) scenario 3. Comparison of the scenarios was implemented using the DIYABC software. The map was drawn using Adobe Illustrator CS5 v15.1.0.

Considering the three scenarios tested with cpDNA sequences, DIYABC analysis software indicated that scenario 2 was the best-supported scenario (Fig. 4), with a posterior probability value higher than those of the other two scenarios (Supplementary Fig. S1). Scenario 2 indicated that *Tamarix* plants in the lower reaches of the Yellow River came from the upstream giving this scenario more support.

### Species distribution modeling

**Figure 5:**
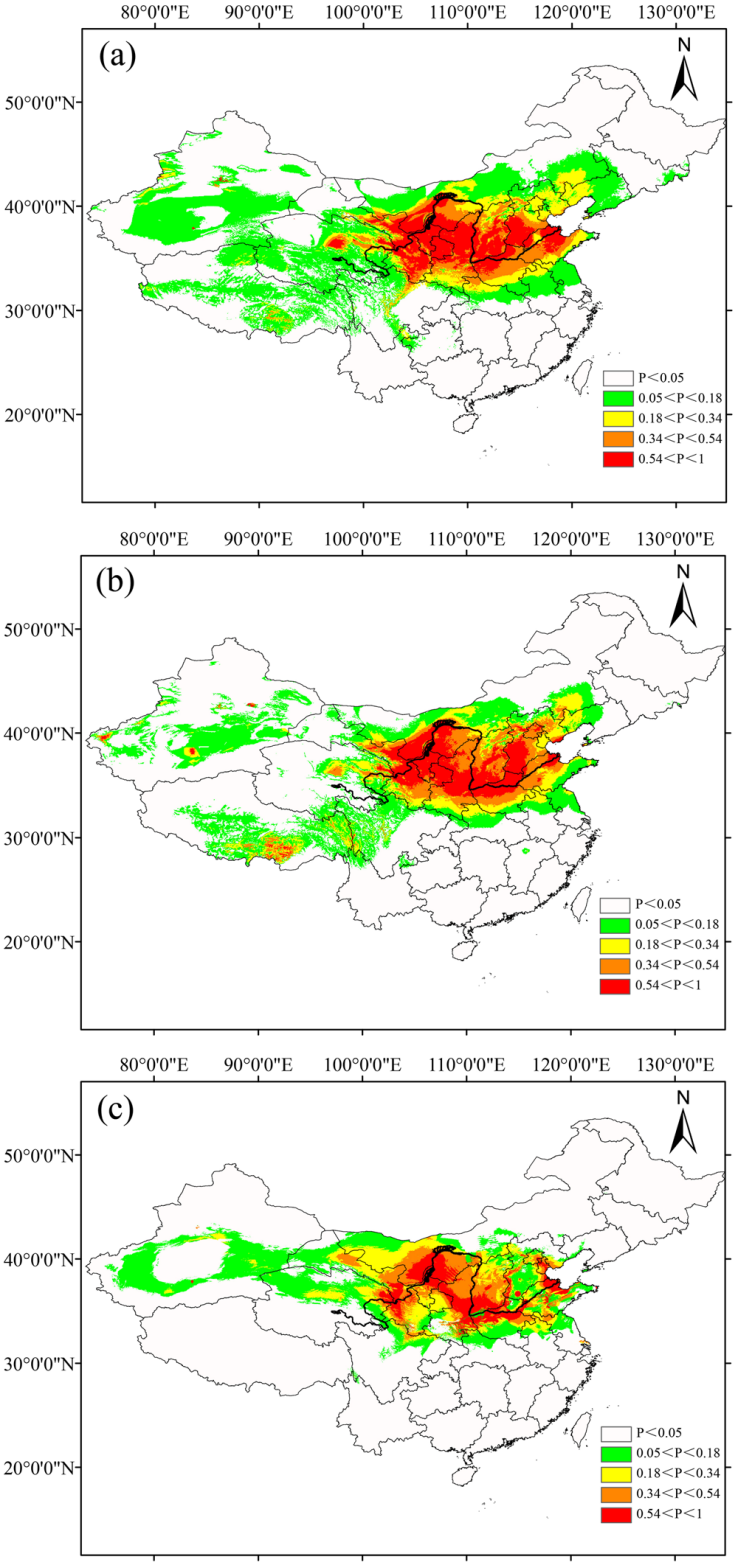
Predictions of suitable habitats for *Tamarix austromongolica* and *Tamarix chinensis* based on ecological niche modeling using MaxEnt. Predicted distributions are shown for (**a**) the present time, (**b**) the last glacial maximum period, about 22,000 years ago, and (**c**) the last interglacial period, 120,000–140,000 years ago. The base map was downloaded from DIVA-GIS. We reclassified the values in the model output as five adaptability levels; high and low values indicate that the conditions are suitable and unsuitable for the species to occur, respectively. The map was drawn using Diva-GIS v7.5.0 and Adobe Illustrator CS5 v15.1.0.

The model algorithm provided the best results based on the area under the receiver operating characteristic curve (AUC) values; AUC values for these curves varied from 0.941 to 0.971. For both species, the most suitable area climatically (0.54 < P < 1) from the current prediction (601,927 km^2^) was obviously larger than that from the last glacial maximum (LGM) prediction (564,288 km^2^) and the last interglacial (LIG, 347,475 km^2^) predictions; similar patterns were identified for all suitable areas (P > 0.05) (Fig. 5a,b and c). Obviously, climatically suitable areas from prediction expanded during the Quaternary period.

Results from the analysis of variable contributions indicated that three environmental variables, namely, mean temperature of coldest quarter, isothermality, and precipitation of wettest month, were the main climatic factors affecting the current distribution of the two *Tamarix* species analyzed here; the percentages of their contributions were 26.8%, 16.7% and 16.4%, respectively. This finding showed that wet habitats and temperate zones were probably the main factors that limit the distribution of *Tamarix* species over time when compared with the effects of other variables.

## Discussion

The most important factors that shape the distribution and demography of populations are natural increases in population size (the number of births minus the number of deaths), net migration, and environmental factors, such as water supply and climate conditions^32–34^. *T. austromongolica* and *T. chinensis* are perennial species with strong tolerance and adaptability; these species blossom and bear fruit 2–3 years after germination. Therefore, both species produce numerous seeds in spring, summer, and autumn. Population growth rates are also influenced by the rates of migration; the small seeds of *Tamarix* species have white hairs on one end that enable long-distance wind or water dispersal^35^, resulting in rapid increases in populations and dispersal along the entire river if environmental conditions are suitable.

The divergence time of C10 in the GYJ population was dated to 1.85 Ma. The paleo-magnetic dating of Liupanshan loess, near the GYJ population, showed the first grade Yellow River terraces reached to 1.8 Ma^36^, and the divergence time of C5 (1.23 Ma) coincided with the Kunlun-Yellow River Tectonic Movement. Therefore, our data provide biological evidence for the approximate geological age of the Yellow River. Based on the divergence time of chlorotype C1 (0.19 Ma), the demographic histories of *T. austromongolica* and *T. chinensis* could be traced back to 0.15 Ma and 0.02 Ma, respectively. As mentioned earlier, the Gonghe Movement started from around 0.15 Ma, which resulted in a substantial uplift of the QTP and promoted the formation of modern Yellow River^37,38^; therefore, it appears that the expansion of these two species was closely matched to the formation of the modern Yellow River.

It is not easy to interpret why the expansion of *T. chinensis* began around 0.02 Ma. *T. austromongolica* and *T. chinensis* are morphologically similar, and both species flower from May to October. Previous studies showed that these two species are closely related phylogenetically^30,31^; therefore, we speculate perhaps *T. chinensis* is a species that evolved and diverged from *T. austromongolica* along the Yellow River, which could explain why the species expanded relatively late. Overall, the valleys of the Yellow River are not only known as cradles of China’s ancient civilization but also provide moist habitat for seed germination and plant growth. Clearly, the Yellow River has been revealed to be the main driving force for demographic expansion of *T. austromongolica* and *T. chinensis*.

These two species experienced demographic expansion during the late Pleistocene, which was a period of multiple glacial-interglacial cycles and dramatic environmental changes. Numerous studies have suggested that environmental changes associated with the Pleistocene climate (e.g., sea-level fluctuations) played roles in determining both the origin and distribution of living organisms^39^, such as mammals^40^, other vertebrates^41^, insects^42^, and plants^43^; however, the responses of different species to these changes have been diverse and depended on their habitat suitability and environmental requirements^44^. For example, neotropical savanna tree species experienced the greatest expansion during the LIG and a retraction during the LGM^45^. Two tropical tree species, *Erythrophleum ivorense* and *E. suaveolens*, experienced demographic bottlenecks during the last glacial period^46^, whereas a temperate tree frog species, *Hyla sarda*, underwent range expansion mostly during the last glacial phase^47^.

*T. austromongolica* and *T. chinensis* are temperate species endemic to China; although this region was not directly affected by extensive ice sheets, it also experienced severe climatic change throughout the Quaternary. Also, during the LGM, the low thermal conditions in the Eurasian continent produced temperatures that were 4 °C–16 °C lower than today^48^. These low temperatures affected the distribution and evolution of plants^49,50^, such as the temperate tree *Pteroceltis tatarinowii*^51^ and the cool-temperate deciduous tree *Quercus mongolica*^52^, which retreated southward and then re-colonized the previously northern region post-glacially. *Ostryopsis davidiana*, which is a temperate deciduous shrub species in northern China, maintained multiple refugia in the northern and southern regions of the Qing Mountains rather than having survived only in the south^53^.

Unlike previous studies, we found that *T. austromongolica* and *T. chinensis* experienced demographic expansion during the late Pleistocene. The EBSP analysis and predictions of paleo-distribution models under past climatic conditions consistently suggested that the populations of these two species not only survived but also experienced a period of range expansion. One interpretation of this is that *Tamarix* is a temperate deciduous species and shows a strong tolerance to cold and drought^54,55^, with killing temperatures for *T. ramosissima*, *T. chinensis*, and hybrids ranging from −33 °C to −47 °C^56^; this type of adaptation was probably important during past range expansions of these populations.

Our genetic data showed that the haplotype diversity value for the entire dataset averaged 0.11, while nucleotide diversity averaged 0.057 × 10^−3^; meanwhile, Hd of the GLZ and NMDK sites were 0.56 and 0.60, respectively, and the populations from the lower reaches seemed to have lower values. Population genetic theory predicts that colonization can result in a decrease in genetic diversity, known as the founder effect, and there is a loss of diversity in newly established populations due to a small number of founders^57–59^.

Various researchers have experimentally investigated the effects of recent colonization events on genetic diversity in both herbs and birds. Recently, D’Andrea *et al*. studied the molecular biogeography of *Lactuca serriola*. They found that significantly lower genetic diversity characterized the newly colonized parts of the range of this species when compared with historical populations; this confirmed the importance of founder effects^60^. Similar results have been reported in *Geranium carolinianum*; genetic diversity patterns across China have revealed that reduced diversity has resulted from successive founder events during range expansion^61^. However, in contrast, long-distance dispersal maximized the evolutionary potential for invasive European starlings (*Sturnus vulgaris*) because multiple introductions associated with successful dispersal strategies may lead to relatively high genetic diversity, especially when introductions occurred from different regions^62^.

Taken together, different introductions and dispersal strategies contributed different amounts of genetic diversity through the introduction of different numbers of individuals or individuals carrying different amounts of diversity. It is well known that genetic diversity is linked to adaptive potential^63^; low genetic diversity in introduced populations may lower their abilities to adapt and hence increase the risk of extinction in novel environments. Thus, to maintain adaptive potential and minimize the risk of extinction, the founder populations with relatively high levels of genetic diversity need to be conserved.

## Conclusions

The main goal of the present study was to understand how the two widespread East Asian temperate deciduous tree species, *T. austromongolica* and *T. chinensis*, responded to climatic fluctuations in the Pleistocene and prehistorical geological events affecting the Yellow River. Based on a wide geographic range of samples and by combining information on cpDNA and nDNA sequences, we dated the divergence time of chlorotypes to the Pleistocene and reconstructed the demographic expansion history of *T. austromongolica* and *T. chinensis* throughout the Yellow River valley based on EBSP, a distribution model, and ABC analyses. The results suggested that the populations of these two species experienced a period of range expansion. Additionally, the occurrence of the expansion of these two species was closely matched to the formation of the modern Yellow River.

## Materials and Methods

### Population sampling

A total of 382 individuals were sampled from 45 populations including 147 individuals of 21 populations of *T. austromongolica* and 235 individuals in 24 populations of *T. chinensis*, during September 2015 to September 2016, across eight provinces along the Yellow River. Of these locations, SFY and SWS were located along the Fenhe River, the second largest tributary of the Yellow River. Additionally, in Shandong Province, *T. chinensis* is common in alkaline and saline soil areas along the coasts of the Bohai Sea; as a result, the sample points here are denser geographically than those of other samples. Moreover, two individuals of *Myricaria bracteata* and *Myricaria paniculata* were collected in Gansu and Qinghai, for use as outgroups in the analysis. Samples of fresh leaves were dried using silica gel; the location of each population, including longitude, latitude and elevation, were recorded with a GPS unit. Table 1 provides detailed information on the sample locations. The voucher specimens were deposited in the herbarium of the Ecological Laboratory of Henan Agricultural University (HEAC).

### DNA extraction, PCR amplification, and sequencing

Total genomic DNA was extracted using a Plant Genomic DNA Extraction Kit (Tiangen, Beijing, China) according to the manufacturer’s protocol and stored at −20 °C. *Trn*L-F^64^, *rps*16^65^ and ITS^66^ were selected; DNA amplifications were performed in a gradient PCR system (Biometra, Germany) with the following cycling conditions: 95 °C (5 min); 38 cycles of 95 °C (30 s), 56 °C (30 s), 72 °C (1 min 50 s), and then 72 °C (10 min); conditions only differed based on the annealing temperatures (58 °C for *rps* 16). We carried out PCR amplification in 20 μL reaction volumes for each individual; PCR products were checked on 1.5% agarose gels and purified with the TaKaRa MiniBEST Agarose Gel DNA Extraction Kit (Dalian, China); all PCR products were subsequently sequenced on an ABI 3730 DNA Sequence Analyzer at the Beijing Genomics Institute (Beijing, China), sequencing with forward and reverse primers in all individuals. Sequences *trn*L-F (KJ729796.1) and *rps*16 (KJ729745.1) of *Reaumuria soongarica* were downloaded from the US National Center for Biotechnology Information (http://www.ncbi.nlm.nih.gov/).

### Nucleotide diversity and genealogy

Sequence contig was done using Seqman with the DNAstar program (DNAstar, Madison, WI, USA); all the variance sites were carefully checked optically in Chromas. For nDNA ITS sequences, if double peaks occurred in the same position and the weakest signal reached 1/3 of the strongest signal, we considered the site to be heterozygous, and inferred their phases using the PHASE algorithm in DnaSP 5.10^67^. Haplotype diversity (Hd) and nucleotide diversity (π) inferred from cpDNA sequences within populations were all done in DnaSP. The genealogical topologies of chlorotypes and ribotypes were constructed using the program Network Version 5.0 with a median-joining model.

### Genetic structure

To determine whether the populations were structured, genetic differentiation among and within populations was calculated using STRUCTURE 2.3.4^68^ and Arlequin ver3.5^69^; the significance of AMOVA was tested based on 1000 permutations.

### Divergence time of chlorotypes

Regarding the fossil record of *Tam*arix, Kräusel^70^ believed that *Tamarix* records in Egypt were from the Lower Oligocene. Researchers in China^71,72^ found *Tamarix* fossils from Gansu Province dated to the Oligocene Baiyanghe Fm and Huoshaogou Fm; all these showed the *Tamarix* plant appeared before the Oligocene. Tamaricaceae and Frankeniaceae formed the closest sister clade with this node dated to 43–30 Ma^73^; Zhang *et al*. estimated an age of about 70 Ma in the light of woody families origin^74^. Tank *et al*. suggested an age for this clade of 49.7 Ma^75^ and ca 53.8 Ma in Magallón *et al*.^76^. Ultimately, 53.8 Ma was chosen cautiously as the family root for our molecular dating.

To relate genetic differentiation found among chlorotypes to Pleistocene events, the divergence time was estimated using BEAST Version 2.3.2^77^; the best fit nucleotide substitution model (GTR+G) was selected with Modeltest Version 3.7^78^ in conjunction with PAUP* Version 4.0b10^79^, Model selection was conducted based on Bayesian Information Criterion. Relaxed clock log-normal was implemented and the mutation rate of 0.9 × 10^−9^ substitutions per site per year for cpDNA noncoding regions was used to calibrate the tree^80^. We ran the Markov chain Monte Carlo (MCMC) chain for 10 million generations, with a sampling every 1,000 generations. We used Tracer software to visualize and check for convergence to a stationary distribution and for high effective sampling size values (ESSs > 200); the first 10% of trees were discarded as burn-in in TreeAnnotator Version 2.3.2, and the resulting trees were drawn in FigTree Version 1.4.2.

### Demographic history

We used Tajima’s D and Fu’s Fs to infer historical demographic processes. We also conducted mismatch distribution analyses based on both SSDs and the Raggedness index^81^ with parametric bootstrapping (1000 replicates) to estimate population expansion.

To investigate the potentially relatively complex effects of population size dynamics, we retraced the demographic history of *Tamarix* with the EBSPs^82^ in BEAST 2.3.2. We applied a general time-reversible substitution model and strict clock rate 0.9×10^−9^substitutions per site per year; the weights for EBSP operators and the initial value were adjusted to improve MCMC mixing. We used a scale factor of 0.5 for cpDNA because only the female cpDNA contributes to the effective population size, and then the MCMC chain was run for 10,000,000 iterations.

### Evolutionary path of T. austromongolica and T. chinensi

To identify source populations and the colonization patterns of *Tamarix* along the Yellow River, further analysis of the evolutionary paths of *T. austromongolica* and *T. chinensis* was inferred from an ABC using DIYABC software Version 2.1.0^83^. Two groups were defined based on *T. austromongolica* and *T. chinensis* species, and three evolutionary scenarios were developed and tested: (a) scenario 1, in which *T. austromongolica* and *T. chinensis* of size N1 and N2 have diverged t generations in the past from an ancestral population of size N1 + N2; (b) scenario 2, in which *T. austromongolica* derived from ancestral population at t2, *T. chinensis* derived from *T. austromongolica* at t1; (c) scenario 3, in which *T. chinensis* derived from ancestral population at t2, *T. austromongolica* derived from *T. chinensi* at t1. We generated 3, 000 000 simulated datasets per scenario and chose uniform prior distributions for effective population sizes. To confirm the validity of using ABC to analyze our data, a posterior probability of scenario was assessed and finally obtained the best-supported scenario for comparison.

### Species distribution modeling

To predict the area of distribution for *T. austromongolica* and *T. chinensis* in the past climate based on habitat suitability, ecological niche modeling was conducted with the Maximum Entropy algorithm (MaxEnt, ver. 3.3.3k)^84^. Except for our 45 sampling sites, 15 reliable distribution sites were selected for prediction from the National Specimen Information Infrastructure (NSII, www.nsii.org.cn). The latitude and longitude of each *T. austromongolica* and *T. chinensis* collection site in MaxEnt are given in Supplementary Table S3. Nineteen bioclimatic variables of current and LGM (about 22ka, 2.5 arc-minute) and LIG (about120–140 ka, 30 arc-second) were obtained from the Worldclim database (www.worldclim.org); these 19 variables included temperature and precipitation and together are considered particularly pertinent to species distributions. The random test percentage was set to 25%, and the maximum number of iterations was set to 1000. To assess the quality of the generated distribution models, a value representing the AUC was performed.

## Electronic supplementary material

supplementary material
