# Supplementary material for: Demographic expansion of two Tamarix species along the Yellow River caused by geological events and climate change in the Pleistocene
Source: Sci Rep. 2018 Jan 8;8:60. doi: 10.1038/s41598-017-19034-x (PMC5758526; doi:10.1038/s41598-017-19034-x)
Supplement: Supplementary file 1 — supplementary material [file 41598_2017_19034_MOESM1_ESM.pdf]

Demographic expansion of two *Tamarix* species along the Yellow River caused by geological events and climate change in the Pleistocene

Hong-yan Liang[1,2], Zhi-pei Feng[1], Bing Pei[1], Yong Li[1] & Xi-tian Yang[1]

[1]College of Forestry, Henan Agricultural University, Zhengzhou, 450002, China.

[2]Sanmenxia Polytechnic, Sanmenxia, 472000, China.

Correspondence and requests for materials should be addressed to X.-T.Y. (email: xitianyang@aliyun.com)

Table S1 Chloroplast DNA sequence polymorphisms detected in *trn*L-F and *rps*16 of *Tamarix austromongolica* and *Tamarix chinensis*

Chlorotype	Nucleotide position										
	294	329	574	627	643	1021	1229	1326	1329	1436	1449
C1	G	T	A	G	G	G	A	-	G	-	A
C2	.	.	.	.	.	.	.	A	.	-	.
C3	T	.	.	.	.	.	.	-	.	-	.
C4	.	G	.	.	.	.	.	-	.	-	.
C5	.	.	.	.	.	.	.	-	.	-	C
C6	.	.	.	.	.	.	G	-	.	-	.
C7	.	.	.	.	.	A	.	-	.	-	.
C8	.	.	.	.	.	.	.	-	A	-	.
C9	.	.	.	.	.	.	.	-	.	T	.
C10	.	.	C	A	.	.	.	-	.	T	.
C11	.	.	.	.	T	.	.	-	.	-	.

Note: The dots in sequences denote nucleotides are same as C1, “-” denote nucleotide absence.

Table S2 Nuclear DNA sequence polymorphisms detected in ITS regions of *Tamarix austromongolica* and *Tamarix chinensis*

	Nucleotide position																																										
Ribotype	58	174	175	176	220	244	413	470	478	501	529	532	538	548	576	585	593	598	599	600	601	602	611	613	614	617	620	622	625	626	627	632	636	640	643	648	655	658	671	672	674	677	680
R1	G	G	C	A	G	G	G	G	G	C	G	G	G	A	G	C	G	G	G	-	-	-	C	A	G	C	C	T	-	-	-	C	T	T	G	A	C	G	G	C	G	T	G
R2	.	.	.	.	.	.	.	.	.	.	.	.	.	.	.	.	.	.	.	-	-	-	.	G	.	.	.	.	-	-	-	.	.	.	.	.	.	.	.	.	.	.	.
R3	.	.	.	.	.	.	.	.	.	.	.	.	.	.	.	T	.	.	.	-	-	-	.	G	.	.	.	C	-	-	-	.	.	.	.	.	.	.	.	.	.	.	.
R4	.	.	.	.	.	.	.	.	.	.	.	.	A	.	.	T	A	.	.	-	-	-	.	G	.	.	.	.	-	-	-	.	.	.	.	.	.	.	.	.	.	.	.
R5	.	.	.	.	.	.	.	.	.	.	.	.	A	.	.	T	.	.	.	-	-	-	.	G	.	.	.	.	-	-	-	.	.	.	.	.	.	.	.	.	.	.	.
R6	.	.	.	.	.	.	.	.	.	.	.	C	.	.	.	.	.	.	.	-	-	-	.	.	.	.	.	.	-	-	-	.	.	.	.	.	.	.	.	.	.	.	.
R7	.	.	.	.	.	.	.	.	.	.	.	C	.	.	.	T	.	.	.	-	-	-	.	.	.	.	.	.	-	-	-	.	.	.	.	.	.	.	.	.	.	.	.
R8	.	.	.	.	.	.	.	.	.	.	.	.	.	.	.	.	.	.	A	-	-	-	.	G	.	.	.	.	-	-	-	.	.	.	.	.	.	.	.	.	.	.	.
R9	.	.	.	.	.	.	.	.	.	.	.	.	.	.	.	T	.	.	A	-	-	-	.	G	.	.	.	.	-	-	-	.	.	.	.	.	.	.	.	.	.	.	.
R10	.	.	.	.	.	.	.	.	.	.	.	.	.	.	.	T	.	.	.	-	-	-	.	G	.	.	.	.	-	-	-	.	.	.	.	.	.	.	.	.	.	.	.
R11	.	.	.	.	.	.	.	A	.	.	.	.	.	.	.	.	.	.	A	-	-	-	.	G	.	.	.	.	-	-	-	.	.	.	.	.	.	.	.	.	.	.	.
R12	A	.	.	.	.	.	.	.	.	.	.	.	.	.	.	.	.	.	.	-	-	-	.	G	.	.	.	.	-	-	-	.	.	.	.	.	.	.	.	.	.	.	.
R13	A	.	.	.	.	.	.	.	.	.	.	C	.	.	.	.	.	.	.	-	-	-	.	G	.	.	.	.	-	-	-	.	.	.	.	.	.	.	.	.	.	.	.
R14	.	.	.	.	.	.	.	A	.	.	.	.	.	.	.	.	.	.	A	-	-	-	.	G	.	.	.	.	-	-	-	.	.	.	.	.	.	.	.	.	A	.	.
R15	.	.	.	.	.	.	.	.	.	.	.	C	.	.	.	.	.	.	.	-	-	-	.	G	.	.	.	.	-	-	-	.	.	.	.	.	.	.	.	.	.	.	.
R16	.	.	.	.	.	.	.	.	.	.	.	.	.	.	.	.	.	-	-	-	G	G	.	G	.	.	.	.	-	-	-	.	.	.	.	.	.	.	.	.	.	.	.
R17	.	.	.	.	A	.	.	.	.	.	.	.	.	.	.	.	.	.	.	-	-	-	.	G	.	.	.	.	-	-	-	.	.	.	.	.	.	.	.	.	.	.	.
R18	.	.	.	.	.	.	.	.	.	.	.	.	.	.	.	.	.	.	.	-	-	-	.	G	.	.	.	.	-	-	-	.	.	.	.	.	.	A	.	.	.	.	.
R19	.	.	.	.	.	.	.	.	.	.	.	C	.	.	.	T	.	.	.	-	-	-	.	G	.	.	.	.	-	-	-	.	.	.	.	.	.	.	.	.	.	.	.
R20	.	.	.	.	.	.	.	.	.	.	.	.	.	.	.	.	.	.	.	C	G	G	.	G	.	.	G	.	-	-	-	.	.	.	.	.	.	.	.	.	.	.	.
R21	.	.	.	.	.	.	.	.	.	.	.	.	.	.	.	.	.	.	.	C	G	G	G	G	.	.	.	.	-	-	-	.	.	.	.	.	.	.	.	.	.	.	.
R22	.	.	.	.	.	.	.	.	.	.	.	.	.	.	.	.	.	.	.	C	G	G	.	G	.	.	.	.	-	-	-	.	.	.	.	.	.	.	.	.	.	.	.
R23	.	.	.	T	.	.	.	.	.	.	.	.	.	.	.	.	.	.	A	-	-	-	.	G	.	.	.	.	-	-	-	.	.	.	.	.	.	.	.	.	.	.	.
R24	.	.	.	.	.	.	.	.	.	.	.	C	.	.	.	.	.	.	.	-	-	-	.	G	.	.	.	.	-	-	-	.	.	.	.	.	.	.	.	.	A	.	.
R25	.	.	T	.	.	.	.	A	.	.	.	.	.	.	.	.	.	.	A	-	-	-	.	G	.	.	.	.	-	-	-	.	.	.	.	.	.	.	.	.	.	.	.
R26	.	.	.	.	.	.	.	.	.	.	.	.	.	.	.	.	.	.	A	-	-	-	.	G	.	.	.	.	-	-	-	.	.	.	.	.	.	.	.	.	A	.	.

R27	.	.	.	.	.	.	.	A	.	.	.	.	.	.	.	.	.	.	.	-	-	-	.	G	.	.	.	.	-	-	-	.	.	.	.	.	.	.	.	.	.	.	.
R28	.	.	.	.	.	.	.	A	.	.	.	.	.	.	.	T	.	.	.	-	-	-	.	G	.	.	.	.	-	-	-	.	.	.	.	.	.	.	.	.	.	.	.
R29	.	.	.	.	.	.	.	.	.	T	.	.	.	.	.	.	.	.	.	-	-	-	.	G	.	.	.	.	-	-	-	.	.	.	.	.	.	.	.	.	.	.	.
R30	.	.	.	.	.	.	.	.	.	T	.	.	.	.	.	T	.	.	.	-	-	-	.	G	.	.	.	.	-	-	-	.	.	.	.	.	.	.	.	.	.	.	.
R31	.	.	.	.	.	.	.	.	.	.	.	.	.	.	.	.	.	.	.	-	-	-	G	G	C	G	.	.	T	G	C	.	.	.	A	.	.	.	.	-	.	.	.
R32	.	.	.	.	.	.	.	.	.	.	.	.	.	C	.	.	.	.	.	-	-	-	G	G	C	G	.	.	T	G	C	.	.	.	A	.	.	.	.	-	.	.	.
R33	.	.	.	.	.	.	.	.	.	T	.	.	.	.	.	.	.	.	A	-	-	-	.	G	.	.	.	.	-	-	-	.	.	.	.	.	.	.	.	.	.	.	.
R34	.	.	.	.	.	.	.	.	.	.	.	C	.	.	.	.	.	.	A	-	-	-	.	G	.	.	.	.	-	-	-	.	.	.	.	.	.	.	.	.	.	.	.
R35	.	.	.	.	.	.	.	.	.	.	.	.	.	.	A	.	.	.	.	-	-	-	.	G	.	.	.	.	-	-	-	.	.	.	.	.	.	.	.	.	.	.	.
R36	.	.	.	.	.	.	.	.	.	.	.	.	.	.	.	.	.	.	A	-	-	-	.	G	.	.	.	.	-	-	-	.	.	.	A	.	.	.	.	.	.	.	.
R37	.	.	.	.	.	.	.	A	.	.	.	.	.	.	.	T	.	.	A	-	-	-	.	G	.	.	.	.	-	-	-	.	.	.	.	.	.	.	.	.	.	.	.
R38	.	.	.	.	.	.	.	.	.	.	.	.	.	.	.	T	.	.	A	-	-	-	.	G	.	.	.	.	-	-	-	.	.	.	.	.	.	.	.	.	A	.	.
R39	.	.	.	.	.	.	.	.	.	.	.	.	.	G	.	T	.	.	.	-	-	-	.	G	.	.	.	.	-	-	-	.	.	.	.	.	.	.	.	.	.	.	.
R40	.	.	.	.	.	.	.	.	A	.	.	C	.	.	.	.	.	.	A	-	-	-	.	G	.	.	.	.	-	-	-	.	.	.	.	.	.	.	.	.	.	.	.
R41	.	.	.	.	.	.	.	.	.	.	.	.	-	.	.	T	.	.	.	-	-	-	.	G	.	.	.	.	-	-	-	.	.	.	.	.	.	.	.	.	.	.	.
R42	.	A	.	.	.	.	.	.	.	.	.	.	-	.	.	T	.	.	.	-	-	-	.	G	.	.	.	.	-	-	-	.	.	.	.	.	.	.	.	.	.	.	.
R43	.	.	.	.	.	.	.	.	.	.	.	.	.	.	.	.	.	.	.	-	-	-	.	G	.	.	.	.	-	-	-	T	C	G	.	G	G	.	T	.	.	G	T
R44	.	A	.	.	.	.	.	.	.	.	.	.	A	.	.	T	.	.	.	-	-	-	.	G	.	.	.	.	-	-	-	.	.	.	.	.	.	.	.	.	.	.	.
R45	.	.	.	.	.	.	.	.	.	.	.	.	.	.	.	.	.	.	.	-	-	-	.	G	.	.	.	.	-	-	-	.	A	.	.	.	.	.	.	.	.	.	.
R46	.	.	.	.	.	.	.	.	.	.	.	.	.	.	A	.	.	.	A	-	-	-	.	G	.	.	.	.	-	-	-	.	.	.	.	.	.	.	.	.	.	.	.
R47	.	.	.	.	.	.	.	.	.	.	.	.	.	.	A	.	.	.	A	-	-	-	.	G	.	.	.	.	-	-	-	.	.	.	A	.	.	.	.	.	.	.	.
R48	.	.	.	.	.	.	.	.	.	.	.	.	.	.	.	.	.	.	.	-	-	-	.	G	.	.	.	.	-	-	-	.	.	G	.	.	.	.	T	.	.	.	.
R49	.	A	.	.	.	.	.	.	.	.	.	.	.	.	.	.	.	.	.	-	-	-	.	G	.	.	.	.	-	-	-	.	.	.	.	.	.	.	.	.	.	.	.
R50	.	.	.	.	.	.	.	.	.	.	A	.	.	.	.	.	.	.	.	-	-	-	.	G	.	.	.	.	-	-	-	.	.	.	.	.	.	.	.	.	.	.	.
R51	.	.	.	.	.	.	A	.	.	.	.	.	.	.	.	T	.	.	.	-	-	-	.	G	.	.	.	.	-	-	-	.	.	.	.	.	.	.	.	.	.	.	.
R52	.	.	.	.	.	.	.	.	.	.	.	C	A	.	.	T	.	.	.	-	-	-	.	G	.	.	.	.	-	-	-	.	.	.	.	.	.	.	.	.	.	.	.
R53	.	A	.	.	.	.	.	.	.	.	.	C	A	.	.	T	.	.	.	-	-	-	.	G	.	.	.	.	-	-	-	.	.	.	.	.	.	.	.	.	.	.	.
R54	.	.	.	.	.	.	.	.	.	.	.	C	A	.	.	T	T	.	.	-	-	-	.	G	.	.	.	.	-	-	-	.	.	.	.	.	.	.	.	.	.	.	.
R55	.	A	.	.	.	.	.	.	.	.	.	C	A	.	.	T	T	.	.	-	-	-	.	G	.	.	.	.	-	-	-	.	.	.	.	.	.	.	.	.	.	.	.
R56	.	.	.	.	.	A	.	.	.	.	.	.	.	.	.	T	.	.	.	-	-	-	.	G	.	.	.	.	-	-	-	.	.	.	.	.	.	.	.	.	.	.	.

Note: The dots in sequences denote nucleotides are same as R1, "-" denote nucleotide absence.

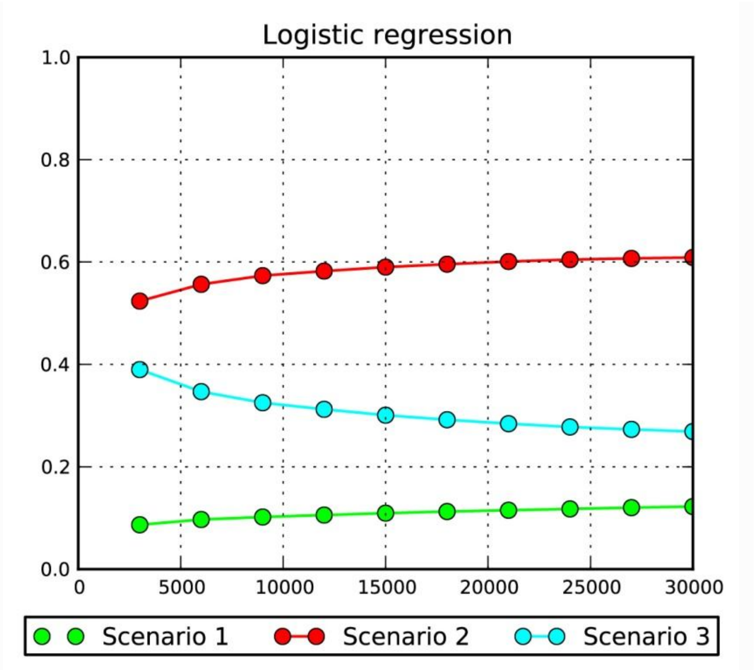

Fig. S1 A comparison of population histories in *Tamarix austromongolica* and *Tamarix chinensis*. Scenario 2 was the best-supported scenario.

Table S3 The latitude and longitude of each *Tamarix austromongolica* and *Tamarix chinensis* collection site in MaxEnt

No.	species	long.	lat.	No.	species	long.	lat.
1	*T.austromongolica*	80.72906	37.01607	31	*T.austromongolica*	111.1425	39.30427
2	*T.austromongolica*	110.2682	37.50391	32	*T.austromongolica*	111.0653	39.22317
3	*T.austromongolica*	107.0128	40.32835	33	*T.austromongolica*	111.0348	39.01114
4	*T.austromongolica*	107.0033	40.42302	34	*T.austromongolica*	112.1456	37.27298
5	*T.austromongolica*	105.6811	37.49115	35	*T.austromongolica*	109.1025	37.21218
6	*T.austromongolica*	91.59592	37.58032	36	*T.austromongolica*	110.4152	37.26446
7	*T.austromongolica*	106.2445	38.54427	37	*T.chinensis*	112.1138	37.06045
8	*T.austromongolica*	99.07666	36.79112	38	*T.chinensis*	110.3626	35.39386
9	*T.austromongolica*	101.3911	36.0344	39	*T.chinensis*	110.2034	35.09419
10	*T.austromongolica*	102.7981	36.32229	40	*T.chinensis*	110.1519	34.50176
11	*T.austromongolica*	101.9428	36.84065	41	*T.chinensis*	110.1351	34.45021
12	*T.austromongolica*	102.6757	36.40228	42	*T.chinensis*	110.172	34.36312
13	*T.austromongolica*	83.6558	37.93014	43	*T.chinensis*	111.202	34.50224
14	*T.austromongolica*	106.373	38.10072	44	*T.chinensis*	111.2047	34.493
15	*T.austromongolica*	104.7856	33.43897	45	*T.chinensis*	111.2047	34.493
16	*T.austromongolica*	102.5508	35.51492	46	*T.chinensis*	111.1636	34.47581
17	*T.austromongolica*	102.5716	36.12249	47	*T.chinensis*	113.0558	34.48496
18	*T.austromongolica*	103.1448	35.57266	48	*T.chinensis*	113.3014	34.57382
19	*T.austromongolica*	103.4823	36.0358	49	*T.chinensis*	113.3905	34.54528
20	*T.austromongolica*	104.4238	36.35306	50	*T.chinensis*	114.2117	34.54286
21	*T.austromongolica*	107.0459	37.31267	51	*T.chinensis*	115.4139	34.3441
22	*T.austromongolica*	106.0909	37.59472	52	*T.chinensis*	117.0019	36.43233
23	*T.austromongolica*	106.3356	38.30052	53	*T.chinensis*	117.544	37.51296
24	*T.austromongolica*	106.4658	39.13509	54	*T.chinensis*	118.0107	37.55093
25	*T.austromongolica*	106.4637	39.39218	55	*T.chinensis*	118.0412	37.44112
26	*T.austromongolica*	107.0145	40.18515	56	*T.chinensis*	118.4318	38.01127
27	*T.austromongolica*	107.2203	40.40283	57	*T.chinensis*	118.4056	38.03315
28	*T.austromongolica*	109.5341	40.18027	58	*T.chinensis*	118.4318	37.53118
29	*T.austromongolica*	110.3855	40.12251	59	*T.chinensis*	118.3226	37.36477
30	*T.austromongolica*	111.1242	40.11176	60	*T.chinensis*	119.0912	37.45334
